# Current status of community-acquired infection of COVID-19 in delivery facilities in Japan

**DOI:** 10.1371/journal.pone.0251434

**Published:** 2021-05-20

**Authors:** Junichi Hasegawa, Tatsuya Arakaki, Akihiko Sekizawa, Tomoaki Ikeda, Isamu Ishiwata, Katsuyuki Kinoshita, on behalf of department of medical safety, the Japan Association of Obstetricians and Gynecologists (JAOG)

**Affiliations:** 1 Department of Obstetrics and Gynecology, St. Marianna University School of Medicine, Kawasaki, Kanagawa, Japan; 2 Department of Obstetrics and Gynecology, Showa University School of Medicine, Tokyo, Japan; 3 Department of Obstetrics and Gynecology, Mie University School of Medicine, Tsu, Mie, Japan; 4 Ishiwata Obstetrics and Gynecology Hospital, Ibaraki, Japan; 5 Seijyo Kinoshita Hospital, Tokyo, Japan; Xavier Bichat Medical School, INSERM-CNRS - Université Paris Diderot, FRANCE

## Abstract

A nationwide questionnaire survey about community-acquired infection of coronavirus disease 2019 (COVID-19) was conducted in July 2020 to identify the characteristics of and measures taken by Japanese medical facilities providing maternity services. A case-control study was conducted by including medical facilities with (Cases) and without (Control) community-acquired infection of COVID-19. Responses from 711 hospitals and 707 private clinics were assessed (72% of all hospital and 59% all private clinics provided maternity service in Japan). Seventy-five COVID-19-positive pregnant women were treated in 52 facilities. Community-acquired infection was reported in 4.1% of the facilities. Of these, 95% occurred in the hospital. Nine patients developed a community-acquired infection in the maternity ward or obstetric department. Variables that associated with community-acquired infection of COVID-19 (adjusted odds ratio [95% confidence interval]) were found to be state of emergency prefecture (4.93 [2.17–11.16]), PCR test for SARS-CoV-2 on admission (2.88 [1.59–5.24]), and facility that cannot treat COVID-19 positive patients (0.34 [0.14–0.82]). In conclusion, community-acquired infection is likely to occur in large hospitals that treat a higher number of patients than private clinics do, regardless of the preventive measures used.

## Introduction

**Fig 1:**
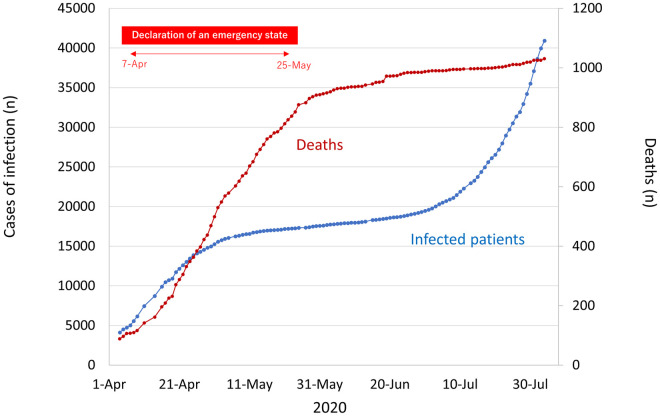
Cumulative number of patients and deaths due to COVID-19 in Japan.

The World Health Organization (WHO) declared the Coronavirus disease 2019 (COVID-19) outbreak a public health emergency of international concern (PHEIC) in January 2020 and a pandemic in March 2020 [1]. In Japan, since the first case of COVID-19 reported on January 16, 2020 [2], the number of domestic infections and deaths have reached over forty-thousand and one thousand, respectively, in August 2020 (Fig 1).

After declaring the COVID-19 pandemic in March, PCR tests for SARS-CoV-2 for examining symptomatic patients and those in close contact with COVID-19-positive individuals were established as a priority. A state of emergency for COVID-19 had also been declared in highly populated prefectures on 7^th^ April 2020. In these areas, the government closed schools, companies, and shops except facilities related to life lines, and requested citizens to stay home. Consequently, the COVID-19 pandemic in Japan was under control until the cancellation of this declaration on 25^th^ June.

The UK Obstetric Surveillance System (UKOSS) demonstrated an estimated incidence of 4.9 COVID-19 cases during pregnancy per 1000 maternities [3]. Despite the increasing number of published studies on COVID-19 in pregnancy, there is currently limited data on the perinatal outcomes of the infection.

A report from a hospital in Wuhan suspected that 41% of patients, and 26% of ICU-patients possibly had hospital-related transmission of COVID-19 [4]. Since COVID-19 can transmit person-to-person and a significant number of carriers are asymptomatic due to low virulence and longer incubation period of the virus, asymptomatic carriers and patients in the incubation period, including health care workers, did not take adequate precautions and thus, became a source of transmission [4–6]. Also, in Japan, community-acquired infections of COVID-19 in the medical facilities have been reported.

In Japan, the maternity service system is slightly different from that in other developed countries. There are more than 2000 maternity facilities that provide delivery services throughout Japan for approximately 860 thousand deliveries per year. More than half of all deliveries are managed in private clinics operated by one or two obstetricians, since pregnant women are more likely to prioritize accessibility and comfort of such a private delivery facility. In these clinics, Polymerase Chain Reactions (PCR) tests, chest computed tomography (CT), and detailed laboratory tests to screen for COVID-19 are usually not available. Thus, when somewhat complications occur during pregnancy pregnant women in private clinic often refer to another hospital.

In such situation in Japan, we hypothesized various characteristics and preventive measures for COVID-19 existed among hospitals and private clinics. Therefore, the Japan Association of Obstetricians and Gynecologists (JAOG) planned a nationwide survey related to COVID-19 cases in medical facilities providing maternity services. The aim of the present study was to identify the characteristics and measures implemented by facilities that have recorded cases of community-acquired infection of COVID-19.

## Methods

### Study design and participants

The nationwide questionnaire survey was conducted in July 2020 as an investigation by the JAOG. The questionnaire was sent to all medical facilities providing maternity services in Japan. The questionnaire, accompanied by a cover letter outlining the aims of the study, was sent via post to the director, chief obstetrician, or consultant of the feto-maternal medicine or delivery facilities. The answers to the questionnaire were thereafter received via the web form.

The questionnaire included questions regarding the number of deliveries in each facility in 2019, the number of treated pregnant women with COVID-19 until June 2020, community-acquired infection of COVID-19, measures during daily clinical practice for general outpatients, measures during daily clinical practice for inpatients whose admission was not due to an infectious disease, and measures during labor for parturients without confirmed or suspected COVID-19.

The subjects were divided into two groups: medical facilities that had recorded community-acquired infection of COVID-19 and control facilities that did not record community-acquired infection of COVID-19. A case-control study was conducted to compare variables associated with clinical characteristics, risk factors, and measures against COVID-19.

### Statistical analysis

The data were entered into a computerized data analysis program (Statistical Package for Social Science (SPSS), Windows version 25.0J; Chicago, IL, USA). Categorical variables were reported as percentages and compared using Fisher’s exact test. The multivariable analyses were based on logistic regression analyses. Significant contributing factors determined by the univariate analyses between medical facilities that experienced community-acquired infection and controls, including facility that cannot treat patients with COVID-19, emergency prefecture, goggle or face shield, PCR test for SARS-CoV-2, patient face mask in the delivery room and limited attendance of birth partners during labor, were used in the multivariable analyses. Statistical significance was defined as a P-value of less than 0.05.

### Ethical approval

This study was approved by the JAOG (No. 80, July. 1st, 2020). This investigation was conducted according to the principles of the Declaration of Helsinki. Informed consent was not obtained from patients or their families because this study was based on the analysis of institutional forms, and the patient records/information was anonymized prior to the analysis.

## Results

**Fig 2:**
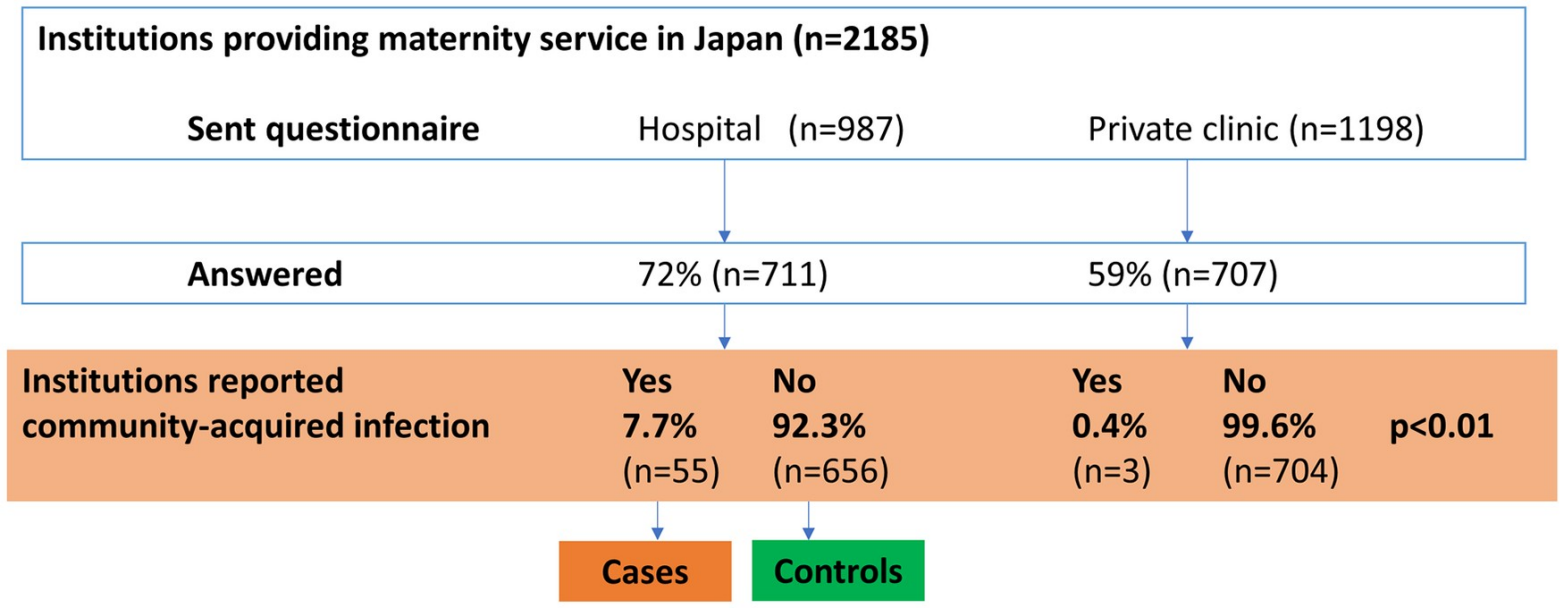
Study flow diagram.

The questionnaire was sent to 2,185 facilities, and responses from 711 hospitals and 707 private clinics were assessed (72% of all hospital and 59% all private clinics provided maternity service in Japan). The study flow diagram is shown in Fig 2. Based on the answers, the total number of deliveries in 2019 among the facilities was 611,444 (71.1% of total deliveries 2019 in Japan; 358,767 in hospitals and 252,677 in private clinics). Of these, seventy-five COVID-19-positive pregnant women were treated in 52 facilities. Number of medical facilities where community-acquired infection of COVID-19 occurred were fifty-five (7.7%) of hospitals and three (0.4%) of private clinics (p<0.01).

Nine facilities were community-acquired infections in the maternity or in the obstetric departments (8 cases in hospitals and a case in private clinic). Six facilities of the community-acquired infection in the inpatient ward, two facilities in the administration office, and one in the obstetric outpatient clinic were reported. The infection route through medical personnel, including doctor, nurse or midwife, was estimated in six cases (66%), whereas infection route through COVID-19-positive patients was reported in only one case. After the occurrence of a community-acquired infection, delivery service was interrupted in 5 facilities and the outpatient clinic was interrupted in two facilities. It took more than two weeks to recover normal practice in these facilities.

**Fig 3:**
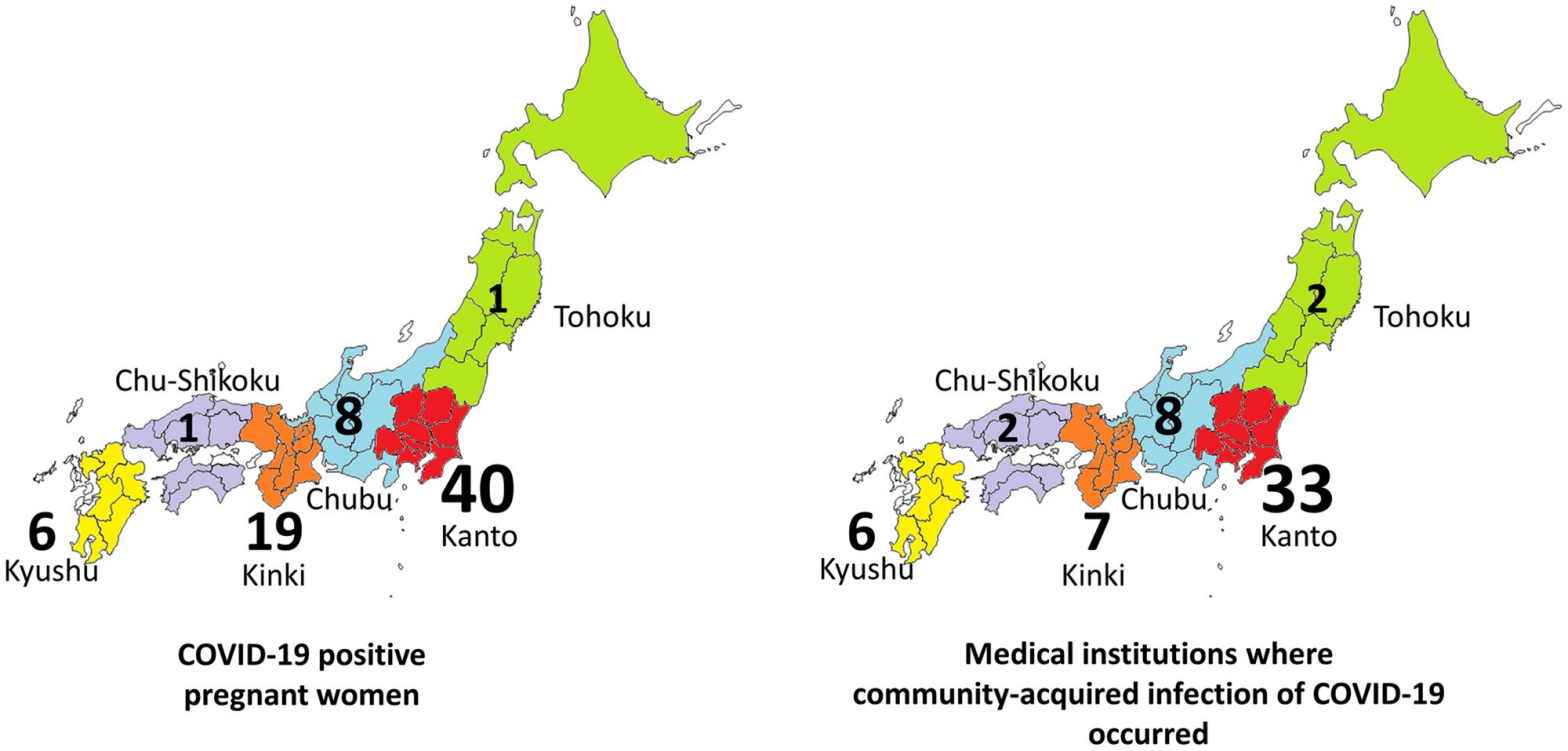
Geographical distribution of pregnant women with COVID-19, and medical facilities where community-acquired infection of COVID-19 occurred in Japan (-2020 June).

Geographical distribution of pregnant women with COVID-19 and medical facilities where community-acquired infection of COVID-19 occurred in Japan until 2020 June are shown in Fig 3. Similar distribution between the incident of COVID-19 positive pregnant women and the incident of community-acquired infection was observed. In prefectures that had declared a state of emergency for COVID-19, 7.2% (39/541) of facilities had reported community-acquired infections. In other prefectures, which did not declare a state of emergency, 2.2% (19/858) of facilities had community-acquired infections.

Characteristics and preventive measures in hospitals and private clinics in Japan is shown in Table 1. Geographical distribution between the hospital and private clinic was not different. COVID-19 positive pregnant women were treated in 71 cases in hospitals and 4 cases in private clinics (p<0.05). Patients with COVID-19 were not treatable in 27% of hospitals and in 81% of private clinics (p<0.05). Therefore, analysis of characteristics and preventive measure associated with community-acquired infection was performed only hospitals.

**Table 1:** Characteristics and preventive measures in hospitals and private clinics in Japan.

	Hospitals		Private clinics		
	n = 711		n = 707		
**Characteristics of facility**					
General hospital with perinatal center	48%	338	n/a		
General hospital	33%	237	n/a		
Other obstetric hospital	19%	136	n/a		
Number of obstetricians working in facility	**5 (3–9)**		**1 (1–2)**		*
Location Tohoku	12%		8%		
Kanto	31%		28%		
Chubu	18%		20%		
Kinki	15%		14%		
Chu-Shikoku	12%		9%		
Kyushu	12%		20%		
State of emergency city for COVID-19	56%		53%		
Number of delivery (2019)	**428 (254–650)**		**320 (200–474)**		*
Number of COVID-19 positive patients	**10%**	71	**1%**	4	*
**Treatable patients**					
Facility that cannot treat patients with COVID-19	**27%**	**192**	**81%**	**574**	*
Maternal transfer to other hospital					
*in pregnant women had a fever*	**20%**	**140**	**58%**	**413**	*
*in pregnant women with fever and respiratory symptom*	**33%**	**238**	**83%**	**586**	*
**Measures during daily clinical practice for outpatients**					
Measurement body temperature	89%	633	84%	594	
Confirmation of symptoms	**86%**	**611**	**78%**	**554**	*
Alcohol disinfection (patients)	**80%**	**567**	**95%**	**671**	*
Face mask (patients)	96%	685	97%	689	
Medical gown	11%	75	12%	86	
Surgical face mask	85%	605	82%	579	
Goggle or face shield	29%	203	29%	208	
Reduction of booking	28%	201	32%	229	
Limited attendance	**72%**	**510**	**91%**	**646**	*
Interruption of mothers class	75%	534	71%	502	
Telemedicine	**47%**	**335**	**19%**	**134**	*
**Measures during daily clinical practice for inpatients**					
Measurement body temperature	**49%**	**351**	**57%**	**406**	*
Confirmation of symptoms	79%	561	80%	564	
Alcohol disinfection (patients)	**71%**	**506**	**90%**	**633**	*
Face mask (patients)	63%	451	69%	489	
Medical gown	13%	94	18%	127	
Surgical face mask	85%	603	82%	578	
Goggle or face shield	25%	179	31%	216	
PCR test for SARS-CoV-2	**17%**	**121**	**2%**	**14**	*
Antigen test for SARS-CoV-2	**3%**	**23**	**0%**	**2**	*
Antibody test for SARS-CoV-2	1%	6	2%	13	
Chest CT	**6%**	**44**	**1%**	**5**	*
Limited attendance	**92%**	**651**	**85%**	**598**	*
**Measures during labor for parturient without confirmed or suspected COVID-19**					
Face mask (patients)	61%	433	65%	462	
Medical gown	64%	452	64%	451	
Surgical face mask	90%	652	84%	597	
Goggle or face shield	**70%**	**499**	**53%**	**374**	*
PCR test for SARS-CoV-2	**17%**	**118**	**2%**	**12**	*
Antigen test for SARS-CoV-2	**3%**	**18**	**1%**	**1**	*
Antibody test for SARS-CoV-2	1%	5	2%	12	
Limited attendance of birth partners during labor	**66%**	**466**	**46%**	**324**	*

Data indicate % and n, or median (quadrantile range).

*; p value<0.05

Characteristics in 55 hospitals where community-acquired infection occurred and 656 hospitals where that did not occur are shown in Table 2. Number of obstetricians working in the hospital, and number of hospitals treated patients with COVID-19 were larger in hospitals where community-acquire infection occurred than in hospital where that did not occur (p<0.05). PCR-test and antigen test for SARS-CoV-2 were more frequently performed in hospitals where community-acquire infection occurred (p<0.05). Goggle or face shield during daily clinical practice for inpatients, and patients’ face mask were also more strictly carried out in hospitals where community-acquire infection occurred (p<0.05).

**Table 2:** Characteristics and preventive measures in hospitals where community-acquired infection occurred and not occurred in Japan.

Community-acquired infection	Not occurred		Occurred		
	n = 656		n = 55		
**Characteristics of facility**					
Perinatal center	40%	262	49%	27	
Emergency prefecture	54%	352	88%	48	*
Number of obstetricians working in facility	**5 (3–9)**		**7 (5–14)**		*
Number of delivery (2019)	423 (251–648)		456 (317–686)		
Facility that cannot treat patients with COVID-19	**28%**	**186**	**11%**	**6**	*
Maternal transfer to other hospital					
*in pregnant women had a fever*	**21%**	**137**	**5%**	**3**	*
*in pregnant women with fever and respiratory symptom*	**35%**	**229**	**16%**	**9**	*
**Measures during daily clinical practice for outpatients**					
Measurement body temperature	89%	582	93%	51	
Confirmation of symptoms	91%	598	96%	53	
Alcohol disinfection (patients)	80%	527	73%	40	
Face mask (patients)	96%	631	98%	54	
Medical gown	10%	68	13%	7	
Surgical face mask	85%	555	91%	50	
Goggle or face shield	28%	184	35%	19	
Reduction of booking	28%	184	31%	17	
Limited attendance	71%	465	82%	45	
Interruption of mothers class	73%	480	80%	44	
Telemedicine	46%	303	58%	32	
**Measures during daily clinical practice for inpatients**					
Measurement body temperature	49%	319	58%	32	
Confirmation of symptoms	79%	515	84%	46	
Alcohol disinfection (patients)	71%	467	71%	39	
Face mask (patients)	62%	407	80%	44	
Medical gown	13%	85	16%	9	
Surgical face mask	85%	556	85%	47	
Goggle or face shield	**24%**	**158**	**38%**	**21**	*
PCR test for SARS-CoV-2	**15%**	**99**	**40%**	**22**	*
Antigen test for SARS-CoV-2	**3%**	**18**	**9%**	**5**	*
Antibody test for SARS-CoV-2	1%	6	0%	0	
Chest CT	**9%**	**57**	**31%**	**17**	*
Limited attendance	91%	596	100%	55	
**Measures during labor for parturient without confirmed or suspected COVID-19**					
Face mask (patients)	**60%**	**60**	**75%**	**41**	*
Medical gown	76%	76	82%	45	
Surgical face mask	90%	90	93%	51	
Goggle or face shield	69%	69	82%	45	
PCR test for SARS-CoV-2	**15%**	**15**	**38%**	**21**	*
Antigen test for SARS-CoV-2	**2%**	**2**	**7%**	**4**	*
Antibody test for SARS-CoV-2	1%	1	0%	0	
Limited attendance of birth partners during labor	**64%**	**64**	**80%**	**44**	*

Data indicate % and n, or median (quadrantile range).

*; p value<0.05 compared to not occurred facilities.

Results of multivariate logistic regression analysis are shown **in** Table 3. Variables that associated with community-acquired infection of COVID-19 (adjusted odds ratio [95% confidence interval]) were found to be state of emergency prefecture (4.93 [2.17–11.16]), PCR test for SARS-CoV-2 on admission (2.88 [1.59–5.24]), and facility that cannot treat COVID-19 positive patients (0.34 [0.14–0.82]).

**Table 3:** Variables associated with community-acquired infection of COVID-19—Result of multivariate logistic regression analysis.

Variables	Adjusted odds ratio	95% confidence interval	
**State of emergency prefecture**	**4.93**	2.17	11.16
**PCR test for SARS-CoV-2 on admission**	**2.88**	1.59	5.24
**Facility that cannot treat COVID-19 positive patients**	**0.34**	0.14	0.82

## Discussion

Community-acquired infection was reported in 4.1% (58) of medical facilities providing maternity services in Japan. Of these, 95% (55/58) were reported in the hospital, which are likely to be in the emergency prefecture and large hospitals where CT and/or PCR test for SARS-CoV-2 were available, while only 5% were reported in maternity private clinics, which are likely to reject medical practices required for symptomatic or suspected COVID-19 cases.

In the maternity ward, person-to-person transmission of COVID-19 was not associated with contact during labor due to the use of personal precaution equipment, including patient’s face mask, medical gown, surgical face mask, and goggle or face shield, regardless of whether medical facilities had experienced community-acquired infections. Strangely enough, limited attendance of birth partners during labor was seen more in medical facilities that reported community-acquired infection rather than in those which did not experience community-acquired infections. A small number of studies have shown that viral isolates were absent in the amniotic fluid, cord blood, breast milk, and neonatal throat swabs in a subset of patients with COVID-19 [7]. So far, standard precautions seem to be effective in labor and delivery rooms for low-risk parturients.

Community-acquired infection in the maternity ward or obstetric department occurred in 9 facilities, and two-thirds of them were associated with the attending doctor, nurse or midwife. This suggests that community-acquired infections of COVID-19 are more likely to originate from asymptomatic patients or medical staff rather than through patients confirmed to be positive for COVID-19. In fact, goggles or face shields were used only in 30% of the inpatients wards and outpatient clinics, though surgical face masks were frequently used. Community-acquired infection associated with COVID-19 might be characterized by unintentional transmission through health care workers from asymptomatic carriers in large hospitals. This tendency is similar to reports from Wuhan, in which asymptomatic carriers and patients did not take adequate precautions and became a source of transmission; consequently, these affected cases included health care workers [4–6].

Interestingly, typical screening tests for COVID-19, like PCR test for SARS-CoV-2 and chest CT, were frequently performed on admission or before delivery in hospitals where community-acquired infection of COVID-19 occurred. This is considered as a confounding factor in large hospitals because these examinations are not available in small hospitals and private clinics. However, multivariate regression analysis revealed that these examinations are independent variables that can increase chances of community-acquired infection. We speculate that overreliance among healthcare workers on negative PCR results in most of the examined patients is weakening the daily prevention measures against infection, despite the low accuracy and sensitivity of the PCR. We suppose that healthcare workers were reassured that PCR and CT-based hospital strategies aided in isolating infected patients into the emergency ward.

We also believe that effective separation of COVID-19 patients from non-infected cases would be the key to success in the prevention of cross-infection [8]. Segregation of medical facilities to isolate symptomatic or suspected COVID-19-positive patients, concomitant with patient’s triage can be a safe and effective way to avoid community-acquired infections. A previous study suggested increasing the use of PCR tests, for SARS-CoV-2, coupled with temporary segregation of high-risk cases into a transitional ward until results are obtained might facilitate the triaging process [8]. We agree with this strategy, but additionally we suggest that healthcare workers should not overtly rely on the PCR results.

This is the first important report to show the current status of community-acquired infection of COVID-19 in delivery facilities in Japan. The limitation of the present study was that the analysis was based on a questionnaire survey that asked for experiences and parameters associated with COVID-19. Causal evaluations between variables and actual community-acquired infections are limited.

## Conclusion

Community-acquired infections of COVID-19 is likely to occur in large hospitals, which treat a large number of patients, compared to that in private clinics, regardless of the preventive measures used. Community-acquired infection can occur even in medical facilities performing screening tests for COVID-19, like chest CT and PCR tests. Daily screening of symptomatic patients and strengthening standard precaution are important to prevent community-acquired infection due to COVID-19.

## Supporting information

S1 File Questionnaire form (Japanese). (DOCX)

S2 File Questionnaire form (English). (DOCX)

S3 File Dataset. (XLSX)

S4 File STROBE checklist. (DOC)
