# Supplementary material for: Current status of community-acquired infection of COVID-19 in delivery facilities in Japan
Source: PLoS One. 2021 May 20;16(5):e0251434. doi: 10.1371/journal.pone.0251434 (PMC8136647; doi:10.1371/journal.pone.0251434)
Supplement: S1 File — (DOCX) [file pone.0251434.s001.docx]

| **番号** | **施設名** |
| --- | --- |


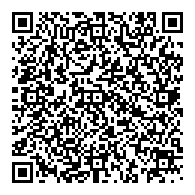
**分娩取り扱い施設における新型コロナウイルス感染症(COVID-19)についての実態調査**

以下の質問では、「COVID-19疑い妊産婦」とは、発熱・呼吸器症状・味覚・臭覚異常・接触歴があり、COVID-19を疑うが、PCR検査未施行、または検査結果が未着な症例を想定しています。

緊急性のある調査のため、できるだけWebでの回答をお願いいたします。
Web回答フォームは、医会ホームページ【ホーム > 産婦人科医会のこと > 部会別資料 > 医療安全部会】からアクセスしてください。
右記QRコードからも回答できます。
FAX利用時（FAX：03-6862-5509）は回答記入した用紙のみ返信ください。

FAX利用の場合は、回答用紙のみの送付をお願いいたします。

【貴施設の感染防止対策等について】

１．COVID-19疑い妊産婦がいた場合のPCR検査は、現在、どこで行いますか？

1. 保健所や帰国者・接触者電話相談センターに相談して決める
2. 連携施設・地域基幹施設に紹介する
3. 自院で検体採取を行い保健所経由でPCR検査を実施する
4. 自院で検体採取とPCR検査を行う
5. その他（　　　　　　　　　　　　　）

２．COVID-19診断確定または疑い妊産婦の診療を貴施設で対応しますか？(複数回答可)

1. 疑いがある患者は対応しない
2. 自院管理中の疑い患者は基本的に対応する
3. 他院からの疑い患者の紹介を基本的に対応する
4. 自院管理中の診断確定患者は基本的に対応する
5. 他院からの診断確定患者も基本的に対応する

３．妊娠末期（37週以降）にCOVID-19の診断確定した妊産婦の分娩方針はどのように決めていますか？

1. 他院に搬送
2. 帝王切開
3. 計画経腟分娩
4. 経過観察（産科的適応に準ず）
5. その他（　　　　　　　　　　　　　）

４．COVID-19を疑う（発熱のみがあり、感染を否定できない）妊産婦が陣痛発来・破水した場合の分娩方針はどのように決めていますか？

1. 他院に搬送
2. 帝王切開
3. 経腟分娩（産科的適応に準ず）
4. その他（　　　　　　　　　　　　　　　）

５．COVID-19を疑う（発熱＋呼吸器症状があり、感染を否定できない）妊産婦が陣痛発来・破水した場合の分娩方針はどのように決めていますか？

1. 他院に搬送
2. 帝王切開
3. 経腟分娩（産科的適応に準ず）
4. その他（　　　　　　　　　　　　　）

６．外来患者による新型コロナウイルスの院内持ち込み対策として、行っている対策はありますか？(複数回答可)

1. 特になし
2. 外来患者の体温測定
3. 外来患者の呼吸器症状などの有無の確認
4. 外来患者の手指アルコール消毒
5. 外来患者のマスク着用
6. 医療者のガウン着用
7. 医療者のサージカルマスク着用
8. 医療者のゴーグル・フェイスシールド着用
9. 医療者の手袋着用
10. 発熱患者の外来受診の停止
11. 外来患者数の抑制・再診患者の診察間隔の延長
12. 外来患者の付き添いの制限
13. 新規初診患者の受け入れ中止
14. 里帰り分娩の制限
15. 母親学級などの中止
16. 産後のメンタルヘルスケアの面談の縮小・中止
17. 電話・Webでの診察と処方箋発行
18. その他（　　　　　　　　　　　　　　　）

７．入院患者による新型コロナウイルスの院内持ち込み対策として、行っている対策はありますか？(複数回答可)

1. 特になし
2. 入院患者の体温測定の頻度の増加
3. 入院患者の呼吸器症状などの有無の確認
4. 入院患者の手指アルコール消毒
5. 入院患者のマスク着用（常時）
6. 入院時（入院前も含む）のPCR検査(SARS-CoV-2)
7. 入院時（入院前も含む）の抗原検査(SARS-CoV-2)
8. 入院時（入院前も含む）の抗体検査(SARS-CoV-2)
9. 医療者のガウン着用
10. 入院時（入院前も含む）の画像検査（胸部X線）
11. 入院時（入院前も含む）の画像検査（胸部CT）
12. 医療者のサージカルマスク着用
13. 医療者のゴーグル・フェイスシールド着用
14. 医療者の手袋着用
15. 発熱患者の緊急入院の停止
16. 良性腫瘍など緊急性のない手術の制限・中止
17. 悪性腫瘍手術の制限・中止
18. 面会制限・中止
19. その他（　　　　　　　　　　　　　　　）

８．通常妊婦の分娩時に行っている感染対策はありますか？(複数回答可)

1. 特になし
2. 産婦のマスク着用（分娩経過中）
3. 入院時（入院前も含む）のPCR検査(SARS-CoV-2)
4. 入院時（入院前も含む）の抗原検査(SARS-CoV-2)
5. 入院時（入院前も含む）の抗体検査(SARS-CoV-2)
6. 医療者のガウン着用
7. 医療者のサージカルマスク着用
8. 医療者のゴーグル・フェイスシールド着用
9. 分娩台へのビニールシートの設置
10. 医療者の手袋着用
11. 立ち合い分娩の中止
12. 家族の来院の制限
13. その他（　　　　　　　　　　　　　　　）

９．貴施設でCOVID-19の院内感染が発生した事実はありますか？(複数回答可)

1. ない **→ 問１８へ**
2. ある

問９でa以外を選択した場合に回答

（施設で院内感染があった場合）

１０．貴施設でCOVID-19の院内感染は？
(複数回答可)

1. 他の診療科で起こった
2. 産婦人科で起こった
3. 医療者間で起こった
4. 病院事務職員間で起こった
5. その他（　　　　　　　　　　　　　　）

１１．院内感染が発生した部署はどこですか？
(複数件の感染が発生した場合は複数回答可)

1. 他診療科の外来
2. 産婦人科外来
3. 他診療科病棟
4. 産婦人科病棟
5. 医局・病院事務など
6. その他（　　　　　　　　　　　　　　）

１２．院内感染の発生原因となった院内へのウイルス持ち込み経路はどのようなものを推定していますか？(複数回答可)

1. 無症候の別疾患の患者
2. 有症状であるが他疾患を疑っていた患者
3. COVID-19を疑っていた患者
4. COVID-19の患者
5. 医師・職員

１３．院内感染の発生をうけ、施設としてどのような対応を実施しましたか？(複数回答可)

1. 関連診療科の外来を停止・制限した
2. 関連診療科の入院を停止・制限した
3. 病院全体の外来診療を停止・制限した
4. 病院全体の新規入院を停止・制限した
5. 入院患者を他施設に紹介した
6. 濃厚接触者のPCR検査を行って隔離（搬送）した
7. その他（　　　　　　　　　　　　　　）

１４．産婦人科内で院内感染があった場合、産科診療でどのような対応をしましたか？

1. 該当しない
2. 産婦人科の外来診療を停止した
3. 入院患者を制限・抑制した
4. 他院からの患者搬送や紹介を停止した
5. 分娩の取り扱いを一時中止(抑制)した(他施設に紹介した)
6. その他（　　　　　　　　　　　　　　）

１５．産婦人科内で院内感染が起こった場合、通常の産科診療に戻るまでにどの程度かかりましたか？

1. 該当しない
2. （　　　　　）日

１６．院内感染の発生によって産科診療において特に困った点や検討が必要だと思った点を記載ください。(自由記載)

１７．院内発生は、後方視的にみて回避可能であったと考えますか？　
可能であると考えた場合は、なにがポイントになると考えますか？ 早期に改善しておくべきであったと考える対応などを記載してください。 (自由記載)

1. いいえ
2. はい　
   （対応）

１８．産婦人科医師や産婦人科内の医療スタッフに感染者はいましたか。また、濃厚接触者は発生しましたか？(複数回答可)

1. いない
2. 感染者がいた　　　
   　⇒　医師（　　　）人；
   　　　医療スタッフ（　　　）人
3. 濃厚接触者がいた　
   　⇒　医師（　　　）人；
   　　　医療スタッフ（　　　）人

１９．貴施設で実施した感染対策のなかで、産科診療を維持するために特に重要であると感じたことを記載してください。 (自由記載)

２０．COVID-19の再流行に備え地域における周産期医療体制を維持するために重要であるとお考えの事項について記載ください。(自由記載)

【貴施設の感染防止対策等について】

２１．貴施設では無症候性の妊産婦に対してSARS-CoV-2のPCR検査（術前実施も含む）を行っていますか？

1. いいえ
2. 実施している・実施した　
   ⇒　検査数：（　　　　）人：
   　　そのうちの陽性者数（　　　　）人

　　6月末までの人数をお答えください

２２．2020年6月末までにCOVID-19疑い（発熱・呼吸器症状・味覚・臭覚異常・接触歴があり、COVID-19を疑ってPCR検査が行われ、結果的には陰性が確認された症例）の妊産褥婦の診療を行いましたか？

1. いいえ
2. いたが、紹介・搬送した　⇒（　　人）
3. 自院で管理した　⇒（　　人）

２３．2020年6月末までにCOVID-19と確定診断された妊産褥婦の診療を行いましたか？

1. いいえ
2. いたが、すぐに紹介した　
   　⇒（　　人）
3. **自院で対応した　
   　⇒（　　人）
   ※【回答用紙２】の記入もお願いします**（全ての症例に関するに調査に協力ください）
