# Supplementary material for: Current status of community-acquired infection of COVID-19 in delivery facilities in Japan
Source: PLoS One. 2021 May 20;16(5):e0251434. doi: 10.1371/journal.pone.0251434 (PMC8136647; doi:10.1371/journal.pone.0251434)
Supplement: S2 File — (DOCX) [file pone.0251434.s002.docx]

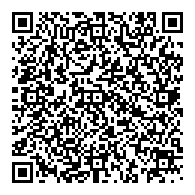
**Survey for COVID-19 in maternity facilities**

Definition of suspicious case with COVID-19: Pregnant women with fever, respiratory symptoms, ageusia, difficult smelling, and/or episode of contact with COVID-19 patient, while result of PCR test had not obtained.

Please, answer to question on this paper or web form.

（FAX：03-6862-5509）

1. Where do you examine PCR test for pregnant women suspected with COVID-19?
2. Health care center
3. Tertiary institution in your region
4. Take specimen in own facility, and send it health care center
5. In own facility
6. Other
7. Can you treat pregnant women with confirmed or suspected COVID-19 in your facility? (multiple choice)
8. Not treatable
9. Treatable for suspected case only in cases taken pregnancy checkups in my hospital.
10. Treatable for all suspected case.
11. Treatable for confirmed case in cases taken pregnancy checkups only in my hospital.
12. Treatable for all confirmed case.
13. How do you treat in pregnant woman confirmed COVID-19 after 37 weeks of gestation?
14. Maternal transfer
15. Cesarean section
16. Planed transvaginal delivery
17. Observation
18. Other
19. How do you treat when pregnant woman with fever without confirmation of COVID-19 occurs rupture of membrane or labor?
20. Maternal transport
21. Cesarean section
22. Transvaginal delivery
23. Other
24. How do you treat when pregnant woman with fever and respiratory symptom without confirmation without confirmation of COVID-19 occurs rupture of membrane or labor?

a. Maternal transport

b. Cesarean section

c. Transvaginal delivery

d. Other

1. Choice preventive measures in your facility during daily clinical practice **for outpatients** (multiple choice)
2. None
3. Thermometry (patients)
4. Confirmation of respiratory symptoms
5. Alcohol disinfection (patients)
6. Face mask (patients)
7. Surgical gown (stuffs)
8. Surgical mask (stuffs)
9. Surgical Goggle or face shield (stuffs)
10. Surgical grove (stuffs)
11. Decline medical care for patients with fever
12. Reduction of booking
13. Limited attendance
14. Decline newly visiting patients
15. Decline reference of patients
16. Interruption of mothers class
17. Reduction of consultation for mothers
18. Telemedicine
19. Other
20. Choice preventive measures in your facility during daily clinical practice **for inpatients** (multiple choice)
21. None
22. Frequent thermometry (patients)
23. Alcohol disinfection (patients)
24. Face mask (patients)
25. PCR teat　(SARS-CoV-2)
26. Antigen test (SARS-CoV-2)
27. Antibody test (SARS-CoV-2)
28. Surgical gown (stuffs)
29. Routine chest XP
30. Routine chest CT
31. Surgical mask (stuffs)
32. Surgical Goggle or face shield (stuffs)
33. Surgical grove (stuffs)
34. Decline inpatient medical care for patients with fever
35. Interruption of operation for benign disease
36. Interruption of operation for malignant disease
37. Limited attendance
38. other
39. Choice preventive measures in your facility during daily clinical practice **at delivery room** (multiple choice)
40. None
41. Face mask (patients)
42. PCR teat　(SARS-CoV-2)
43. Antigen test (SARS-CoV-2)
44. Antibody test (SARS-CoV-2)
45. Surgical gown (stuffs)
46. Surgical mask (stuffs)
47. Surgical Goggle or face shield (stuffs)
48. Curtain shield
49. Surgical grove (stuffs)
50. Decline delivery with partner
51. Limited attendance
52. other
53. Have you experienced community-acquired infection of COVID-19 in your facility?
54. No **→ Go Question 18**
55. Yes
56. Situation of community-acquired infection in your facility (multiple choice)
57. Patients ward in another department
58. Patients ward in OBGY
59. Among medical care givers
60. Among administrative staff
61. other
62. Location of community-acquired infection in your facility (multiple choice)
63. Outpatients ward in another department
64. Outpatients ward in OBGY
65. Inpatients ward in another department
66. Inpatients ward in OBGY
67. other
68. How do you think community-acquired infection occurred from?
69. Asymptomatic patients
70. Symptomatic patients not suspected COVID-19
71. Patients suspected COVID-19
72. Patients confirmed COVID-19
73. Medical care givers or administrative stuffs
74. How did you do in your facility after community-acquired infection?
75. Limited department of outpatients’ clinic associated with infection
76. Limited department of inpatients’ ward associated with infection
77. Interrupted all of outpatients’ medical care
78. Interrupted all of inpatients’ medical care
79. Transferred all inpatients to another hospital
80. Transferred patients with confirmed and suspected COVID-19.
81. Other
82. How did you do in your facility after community-acquired infection in obstetric department?
83. n/a
84. Interrupted pregnancy checkups
85. Limited inpatients
86. Limited reference of patients
87. Limited or interrupted deliveries
88. Other
89. How many days did take it recover ordinary medical care?
90. n/a
91. days
92. How did you feel in obstetric management after community-acquired infection?
93. Retrospectively, do you think community acquired infection in your facility could be avoidable?
94. No
95. Yes
96. Were there any medical care givers who infected COVID-19 in your department? Also, suspected COVID-19?
97. No
98. Yes, there was infected person. 　　　
    　⇒　Doctor ( )
    　　　Midwife or nurse ( )
99. Yes, there was suspected person. 　　　
    　⇒　Doctor ( )
    　　　Midwife or nurse ( )
100. Please, comment about important preventive measures for COVID-19 in obstetric department.
101. Please, comment about important preventive measures for COVID-19 in your region.

**Until June, 2020**

1. Did you examine PCR-test for SARS-CoV-2 in asymptomatic pregnant woman?
2. No
3. Yes　
   ⇒　How many patients?
   　　How many positives?

1. Did you examine PCR-test for SARS-CoV-2 in symptomatic pregnant woman but result was negative?
2. No
3. Yes
4. Did you treat pregnant woman with confirmed COVID-19?
5. No
6. Yes, but I referred to another hospital.
7. **Yes, I treat patients in my facility.**
